# Effect of chronic nitrate and citrulline supplementation on vascular function and exercise performance in older individuals

**DOI:** 10.18632/aging.101984

**Published:** 2019-05-29

**Authors:** Thibault Le Roux-Mallouf, Felix Pelen, Angela Vallejo, Idir Halimaoui, Stéphane Doutreleau, Samuel Verges

**Affiliations:** 1 Université Grenoble Alpes, Inserm, HP2 Laboratory, Grenoble F-38000, France; 2 Sport and Pathologies Unit, Grenoble Alpes University Hospital, Hôpital Michallon, Grenoble F-38042, France

**Keywords:** nitric oxide, nitrate, citrulline, exercise, ageing

## Abstract

Increased nitric oxide (NO) bioavailability may improve exercise performance and vascular function. It remains unclear whether older adults who experience a decreased NO bioavailability may benefit from chronic NO precursor supplementation. This randomised, double-blind, trial aims to assess the effect of chronic NO precursor intake on vascular function and exercise performance in older adults (60-70 years old). Twenty-four healthy older adults (12 females) performed vascular function assessment and both local (knee extensions) and whole-body (incremental cycling) exercise tests to exhaustion before and after one month of daily intake of a placebo (PLA) or a nitrate-rich salad and citrulline (N+C, 520mg nitrate and 6g citrulline) drink. Arterial blood pressure (BP) and stiffness, post-ischemic, hypercapnic and hypoxic vascular responses were evaluated. Prefrontal cortex and quadriceps oxygenation was monitored by near-infrared spectroscopy. N+C supplementation reduced mean BP (-3.3mmHg; p=0.047) without altering other parameters of vascular function and oxygenation kinetics. N+C supplementation reduced heart rate and oxygen consumption during submaximal cycling and increased maximal power output by 5.2% (p<0.05), but had no effect on knee extension exercise performance. These results suggest that chronic NO precursor supplementation in healthy older individuals can reduce resting BP and increase cycling performance by improving cardiorespiratory responses.

## Introduction

Nitric oxide (NO) is a gaseous signalling molecule involved in a variety of physiological functions throughout the body [1]. The first pathway for NO production is endogenous via the citrulline-arginine-NO pathway requiring the activity of the nitric oxide synthase (NOS) enzymes. The second pathway is partially exogenous since it uses nitrate and nitrite brought by water and food to produce NO based on the simple one-electron reduction of nitrite. Systemic NO bioavailability can be enhanced by NO precursor supplementation such as arginine [2] and nitrate [3]. Interestingly, it has been shown that oral citrulline supplementation increases the circulating [4,5] and tissue [6] arginine concentration more efficiently than an equivalent dose of arginine, suggesting that exogenous citrulline administration might represent an interesting option to increase the amount of arginine to be converted by NOS in NO.

In the peripheral vessels, NO regulates vascular tone by activating soluble guanylate cyclase in the vascular smooth muscle. During physical activity, NO bioavailability is important to match blood flow to oxygen demand in the brain and contracting muscles. During intermittent handgrip exercise for instance, NOS inhibition via NG-monomethyl-Arginine reduces muscle blood flow [7] and total vasodilator responses to muscle contraction [8]. NO is also an important neurotransmitter and neuromodulator (chemical messenger [9],). It is involved in cerebral blood ﬂow auto-regulation [10] and neurovascular coupling [11,12].

A reduction in NO bioavailability has been singled out as the main cause of endothelial dysfunction [13]. The latter is recognized as an important predictive factor for several cardiovascular disorders and has been implicated in the pathogenesis of hypertension, atherosclerosis, arterial thrombosis [14–16]. Advanced age is associated with endothelium dysfunction due to impairments in NO signalling pathways. Several possible mechanisms may underlie this impairment in NO metabolism, including limited substrate (arginine [17],) and cofactor bioavailability (e.g. tetrahydrobiopterin [18],) and reduced abundance or activity of NOS. In addition to vascular function, cardiorespiratory exercise responses are also considered as a reliable predictive factor for cardiovascular diseases [19,20]. Hence, some recent studies investigated the potential benefits of NO precursor supplementation on vascular function and exercise performance in the older population. Contrasting effects of chronic NO precursor (i.e. arginine, citrulline, nitrate) intake on exercise performances have been reported in older adults [for a review, see [21,22]]. While some studies found a positive effect of nitrate intake on exercise time to exhaustion [23,24] and oxygen consumption (VO_2_) response time [25], other authors showed no significant effect on exercise performance [26]. Some studies have also shown positive vascular effects in older adults following acute and chronic nitrate intake, including reduced blood pressure (BP) [27,28], improved regional brain perfusion [29] and improvements in several parameters of vascular function [30]. However, Miller et al. [31] showed no effect of nitrate supplementation on blood pressure ( BP) despite increased plasma nitrate and nitrite. Regarding citrulline intake, while chronic supplementation has been shown to reduce BP [32], acute ingestion showed no effect on vascular function in older adults with heart failure [33]. These contrasting results may be due to different types of supplementation (i.e. NOS-independent or NOS-dependent supplementation), dosage or duration of supplementation, and health status of participants, making the potential interest and optimal strategy for NO precursor supplementation in older individuals still unclear.

Thus, this study aims to assess the effect of chronic NO precursor supplementation on vascular function, muscle and cerebral oxygenation and performance during both local and whole-body exercise in healthy older adults. To enhance NO bioavailability, nitrate and citrulline supplementation (N+C) were used in order to supplement both NOS-independent and NOS-dependent pathways, since ageing may impair NO bioavailability due to both an impairment in NOS activity and a lack of NOS substrate. We hypothesized that chronic NO precursor intake would improve vascular function and cerebral and muscular responses to exercise, leading to increased exercise performances.

## RESULTS

### Vascular function

**Figure 1:**
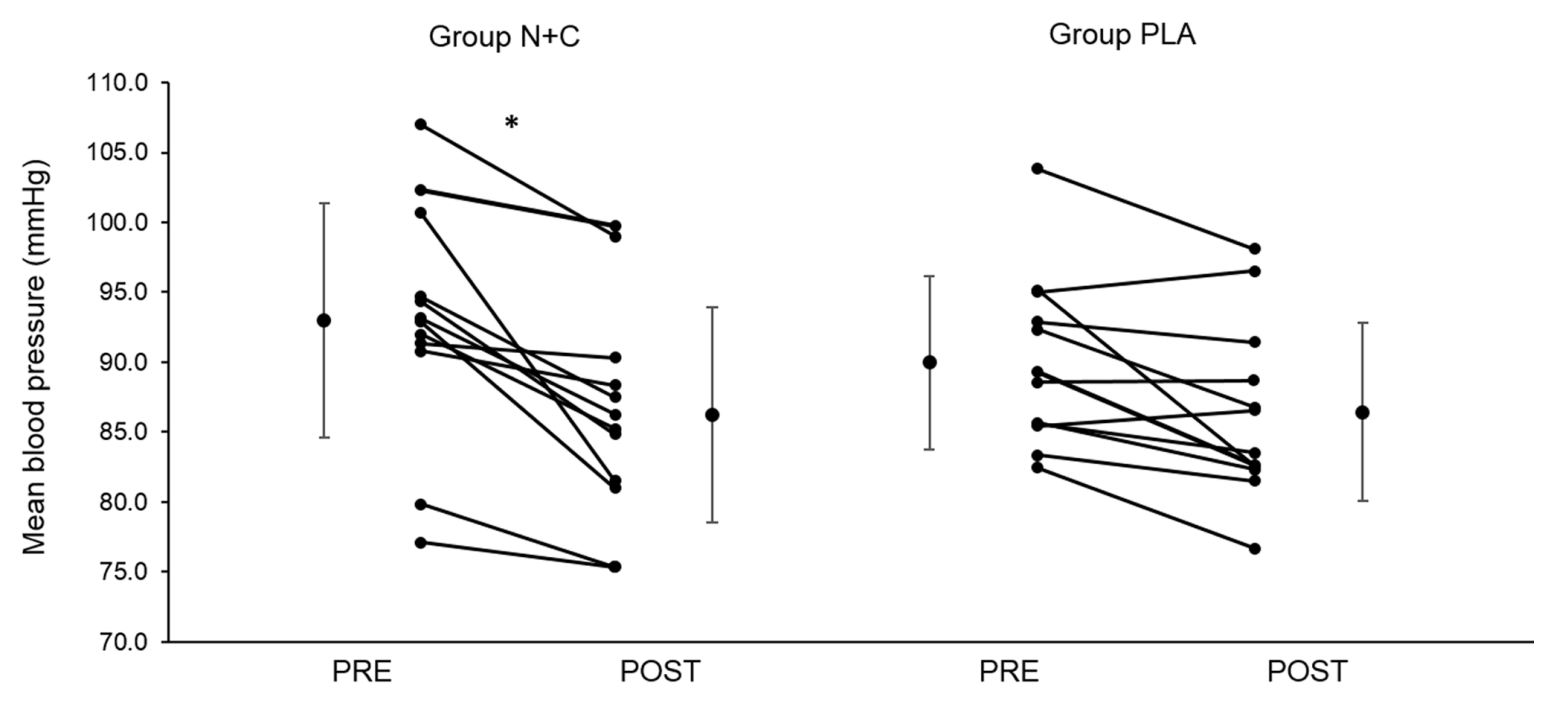
**Individual and group mean changes in mean arterial blood pressure before and after one month of placebo or nitrate and citrulline intake in older adults**. N+C, nitrate + citrulline; PLA, placebo; PRE, measure before the supplementation period; POST, measure after the supplementation period; * significant difference between PRE and POST; n=24.

Resting vascular function parameters are provided in Table 1. There were no significant difference between groups for baseline systolic (SBP), diastolic (DBP), and mean (MBP) blood pressure (all p > 0.05). After one month of supplementation, systolic (SBP) and diastolic (DBP) blood pressure did not change significantly although the PRE-POST difference in SBP tended to be larger in the N+C group compared to placebo (PLA) (N+C versus PLA t-test p value = 0.058, Cohen’s d = 0.660). As shown in Figure 1, the N+C group showed a significantly greater reduction in MBP compared to PLA (p = 0.047, d = 0.71).

**Table 1:** Vascular function before and after one month of NO precursor supplementation.

		**PRE**			**POST**			**∆PRE/POST**			**p∆**	**d**∆
**SBP** (mmHg)	**N+C**	123.2	±	13.9	115.7	±	12.3	-7.5	±	6.5	0.058	0.660
	**PLA**	117.8	±	7.2	114.3	±	8.9	-3.4	±	5.7		
**DBP** (mmHg)	**N+C**	78.2	±	6.5	71.9	±	5.8	-6.2	±	5.1	0.130	0.460
	**PLA**	76.0	±	9.8	72.1	±	6.9	-3.9	±	4.6		
**PWV** (m·s^-1^)	**N+C**	9.2	±	5.9	7.0	±	2.8	-2.2	±	5.3	0.220	0.550
	**PLA**	6.7	±	2.7	6.7	±	3.1	0.0	±	2.1		
**Reperfusion** (mmol of HbO_2_)	**N+C**	15.9	±	11.2	14.4	±	10.3	-1.5	±	3.1	0.250	0.200
	**PLA**	12.7	±	6.7	12.6	±	5.3	-0.7	±	4.9		
**Reperfusion** (mmol of Hbtot)	**N+C**	10.8	±	4.2	10.5	±	6.3	-0.2	±	2.9	0.710	0.130
	**PLA**	9.2	±	4.9	8.4	±	2.3	-0.7	±	4.3		

Data are presented as mean ± SD, n = 24. SBP, systolic blood pressure; DBP, diastolic blood pressure; PWV, carotid-femoral pulse wave velocity; Reperfusion, difference between the value reached at the end of the ischemic phase and the maximal value reached during the reperfusion phase in the ischemia-reperfusion test; HbO_2_, oxyhaemoglobin; HbTot, total haemoglobin. PRE, measure before the supplementation period; POST, measure after the supplementation period. N+C, nitrate + citrulline, PLA, placebo; ∆PRE/POST, difference between PRE and POST measures; p∆, p value for ∆PRE/POST group comparison; d∆, Cohen’s d effect size of N+C supplementation on ∆PRE/POST.

PRE-POST changes in PWV did not differ significantly between groups (all p > 0.05). Similarly, there was no significant difference between groups for post-ischemia ∆max/min HbO_2_ and ∆max/min HbTot (all p > 0.05).

### Hypercapnic and hypoxic responses

As shown in Table 2, there was no significant difference between groups at baseline and no effect of NO precursor on hypercapnic responses at rest (all p > 0.05). As shown in Table 3, there was also no significant difference between groups at baseline and no effect of NO precursor supplementation on hypoxic responses, neither at rest nor during submaximal cycling exercise (all p > 0.05).

**Table 2:** Cardiorespiratory, cerebral and muscle responses to hypercapnia before and after one month of NO precursor supplementation.

			**PRE**			**POST**			**∆PRE/POST**			**p∆**	**d∆**
**∆HR** (bpm)		**N+C**	4.9	±	3.2	2.6	±	5.2	-2.3	±	4.3	0.390	0.120
		**PLA**	2.7	±	3.7	1.0	±	5.3	-1.7	±	6.0		
**∆VE** (l·min^-1^)		**N+C**	18.3	±	9.6	22.3	±	13.9	4.0	±	8.2	0.240	0.290
		**PLA**	15.8	±	9.2	17.5	±	7.2	1.7	±	7.5		
**∆TSI%**	**Cerebral**	**N+C**	2.7	±	4.7	4.3	±	1.9	0.4	±	5.2	0.121	0.710
		**PLA**	6.0	±	3.0	3.5	±	3.6	-2.5	±	2.3		
	**Muscle**	**N+C**	0.9	±	3.4	-0.2	±	3.3	-0.7	±	3.2	0.739	0.296
		**PLA**	1.3	±	4.5	0.0	±	1.7	-1.3	±	4.4		

Data are presented as mean ± SD, n = 24 (n = 23 for TSI). Hypercapnia corresponded to a CO_2_ end tidal partial pressure of +10 mmHg above normoxic level; ∆HR, Heart rate difference between normoxia and hypercapnia; ∆VE, minute ventilation difference between normoxia and hypercapnia; ∆TSI, tissue saturation index difference between normoxia and hypercapnia; N+C, nitrate + citrulline; PLA, placebo; PRE, measure before the supplementation period; POST, measure after the supplementation period. ∆PRE/POST, difference between PRE and POST measures; p∆, p value for ∆PRE/POST group comparison; d∆, Cohen’s d effect size of N+C supplementation on ∆PRE/POST.

**Table 3:** Cardiorespiratory, cerebral and muscle responses to hypoxia at rest and during cycling exercise before and after one month of NO precursor supplementation.

						**PRE**						**POST**						**∆PRE/POST**						**p∆**		**d∆**	
**Rest**	**∆HR** (bpm)		**N+C**		6.6		±	6.5			4.6		±	2.9			-2.6		±	5.9			0.225		0.321	
			**PLA**		5.3		±	4.2			4.8		±	5.2			-0.6		±	6.4						
	**∆VE** (l·min^-1^)		**N+C**		-2.0		±	1.2			-1.5		±	1.4			0.4		±	1.8			0.130		0.470	
			**PLA**		-1.1		±	1.7			-1.6		±	2.1			-0.5		±	2.0						
	**∆SpO_2_** (%)		**N+C**		86.6		±	4.4			86.7		±	4.4			-0.5		±	4.7			0.400		0.110	
			**PLA**		83.9		±	7.9			83.6		±	4.4			0.3		±	9.5						
	**∆TSI%**	**Cerebral**		**N+C**		-4.3			±	7.6		-3.7			±	1.9		-1.5			±	7.5		0.440		0.768	
				**PLA**		-4.4			±	3.2		-3.9			±	2.3		0.5			±	3.1					
		**Muscle**		**N+C**		-0.8			±	2.0		0.2			±	4.3		0.7			±	4.7		0.260		0.959	
				**PLA**		-0.3			±	1.0		-1.4			±	1.7		-1.1			±	1.4					
**Cycling**	**∆HR** (bpm)		**N+C**		10.9		±	9.9			14.3		±	7.6			3.4		±	8.9			0.280		0.240	
			**PLA**		14.8		±	13.8			14.8		±	11.5			-0.1		±	17.4						
	**∆VE** (l·min^-1^)		**N+C**		4.2		±	9.3			6.5		±	6.9			2.4		±	11.2			0.230		0.310	
			**PLA**		5.9		±	9.5			5.2		±	7.2			-0.7		±	8.4						
	**∆SpO_2_** (%)		**N+C**		76.1		±	6.2			77.8		±	9.1			1.7		±	6.7			0.350		0.150	
			**PLA**		73.7		±	9.7			73.9		±	10.5			0.3		±	11.5						
	**∆TSI%**	**Cerebral**		**N+C**		-13.6			±	27.4		-7.6			±	3.3		-1.4			±	10.1		0.198		0.737	
				**PLA**		-8.7			±	6.8		-5.4			±	4.8		3.3			±	4.4					
		**Muscle**		**N+C**		-0.6			±	5.3		0.4			±	10.5		-3.1			±	5.1		0.788		0.393	
				**PLA**		-0.9			±	2.9		-3.5			±	1.9		-2.6			±	3.4					

Data are presented as mean ± SD, n = 24 (n = 23 for TSI). Hypoxia corresponded to an inspiratory oxygen fraction of 11%. ∆HR, heart rate difference between normoxia and hypoxia; ∆VE, minute ventilation difference between normoxia and hypoxia; ∆SpO_2_, pulse oxygen saturation difference between normoxia and hypoxia; ∆TSI, tissue saturation index difference between normoxia and hypoxia; PRE, measure before the supplementation period; POST, measure after the supplementation period; N+C, nitrate + citrulline; PLA, placebo; ∆PRE/POST, difference between PRE and POST measures; p∆, p value for ∆PRE/POST group comparison; d∆, Cohen’s d effect size of N+C supplementation on ∆PRE/POST.

### Knee extension exercise performance

There was no significant difference between groups for TSI (Table 4) and all other NIRS parameters (results not shown; all p > 0.05) during knee extensions. There was also no significant difference between groups regarding PRE-POST changes in MVC and total number of contractions during the knee extension exercise test (p > 0.05, Table 5).

**Table 4:** Tissue saturation index during the cycling test and knee extension test before and after one month of NO precursor supplementation.

				**PRE**						**POST**					
				**50%**			**Exhaustion**			**50%**			**Exhaustion**		
**Cycling**		**Cerebral**	**N+C**	1.9	±	4.8	-4.2	±	3.5	-1.6	±	1.5	-5.3	±	4.9
			**PLA**	-0.1	±	7.5	0.1	±	8.4	-2.3	±	2.5	-5.3	±	3.7
		**Muscle**	**N+C**	-0.9	±	0.5	-1.0	±	2.0	0.9	±	7.8	-1.1	±	3.5
			**PLA**	-1.5	±	0.7	-2.7	±	-3.5	-2.2	±	1.5	3.4	±	1.5
**Knee extension**		**Cerebral**	**N+C**	1.5	±	4.2	-1.9	±	3.7	0.9	±	4.2	-2.1	±	4.2
			**PLA**	-0.6	±	5.0	-3.0	±	4.8	-0.6	±	3.7	-1.8	±	5.7
		**Muscle**	**N+C**	-9.5	±	8.3	-8.3	±	11.6	-9.4	±	6.4	-8.0	±	9.0
			**PLA**	-11.2	±	4.9	-12.5	±	7.4	-12.4	±	7.2	-12.0	±	9.4

Data are presented as mean ± SD changes of tissue saturation index in % from the initial workload (70 W for males and 50 W for females), n = 23. N+C, nitrate + citrulline; PLA, placebo; PRE, measure before the supplementation period; POST, measure after the supplementation period; 50%, 50% of the duration of the PRE test (i.e. isowatt for cycling exercise and isoKg for knee extension exercise). No interaction effect N+C/PLA group × PRE/POST session, all p > 0.05; all Partial eta square < 0.07.

**Table 5:** Performances during the cycling test and the knee extension test before and after one month of NO precursor supplementation.

		**PRE**			**POST**			**∆PRE/POST**			**p∆**	**D∆**
**Maximal power output** (W)	**N+C**	180.9	±	44.3	190.3	±	47.5	9.4	±	11.1	0.021	0.411
	**PLA**	206.0	±	54.5	207.4	±	53.9	1.3	±	7.2		
**VO_2_ max** (ml·kg^-1^·min^-1^)	**N+C**	39.6	±	7.3	40.6	±	6.5	1.2	±	3.8	0.920	0.040
	**PLA**	45.4	±	7.7	46.6	±	7.9	1.4	±	2.8		
**MVC** (Kg)	**N+C**	63.1	±	14.0	65.6	±	18.0	2.5	±	7.6	0.350	0.340
	**PLA**	67.3	±	9.6	65.5	±	12.3	0.2	±	5.5		
**Number of contractions**	**N+C**	149.0	±	44.0	153.0	±	46.0	4.1	±	35.1	0.650	0.020
	**PLA**	161.0	±	33.0	165.0	±	48.0	5.0	±	33.2		

Data are presented as mean ± SD, n = 24. MVC, maximal voluntary contraction; VO_2_max, maximal oxygen consumption; N+C, nitrate + citrulline, PLA, placebo; PRE, measure before the supplementation period; POST, measure after the supplementation period; ∆PRE/POST, difference between PRE and POST measures; p∆, p value for ∆PRE/POST group comparison; d∆, Cohen’s d effect size of N+C supplementation on ∆PRE/POST.

### Incremental cycling exercise test

**Figure 2:**
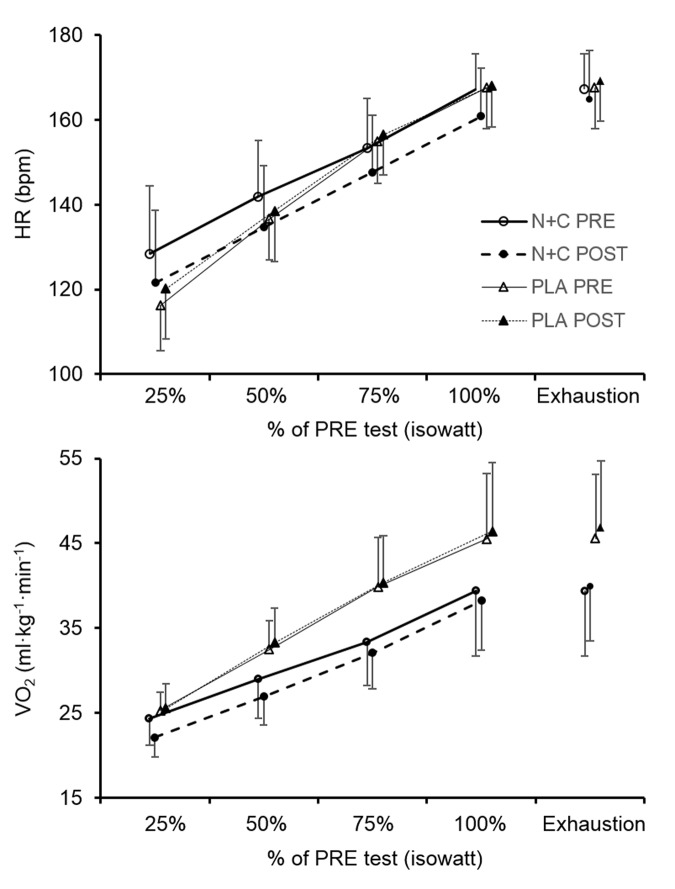
**Heart rate and oxygen consumption during the cycling incremental test before and after one month of placebo or nitrate and citrulline intake in older adults.** HR, heart rate; VO_2_, oxygen consumption; N+C, nitrate + citrulline; PLA, placebo; PRE, measure before the supplementation period; POST, measure after the supplementation period; 25%; 50%; 75%; 100%, 25%, 50%, 75% and 100%, of the duration of the PRE test (i.e. isowatt).

**Figure 3:**
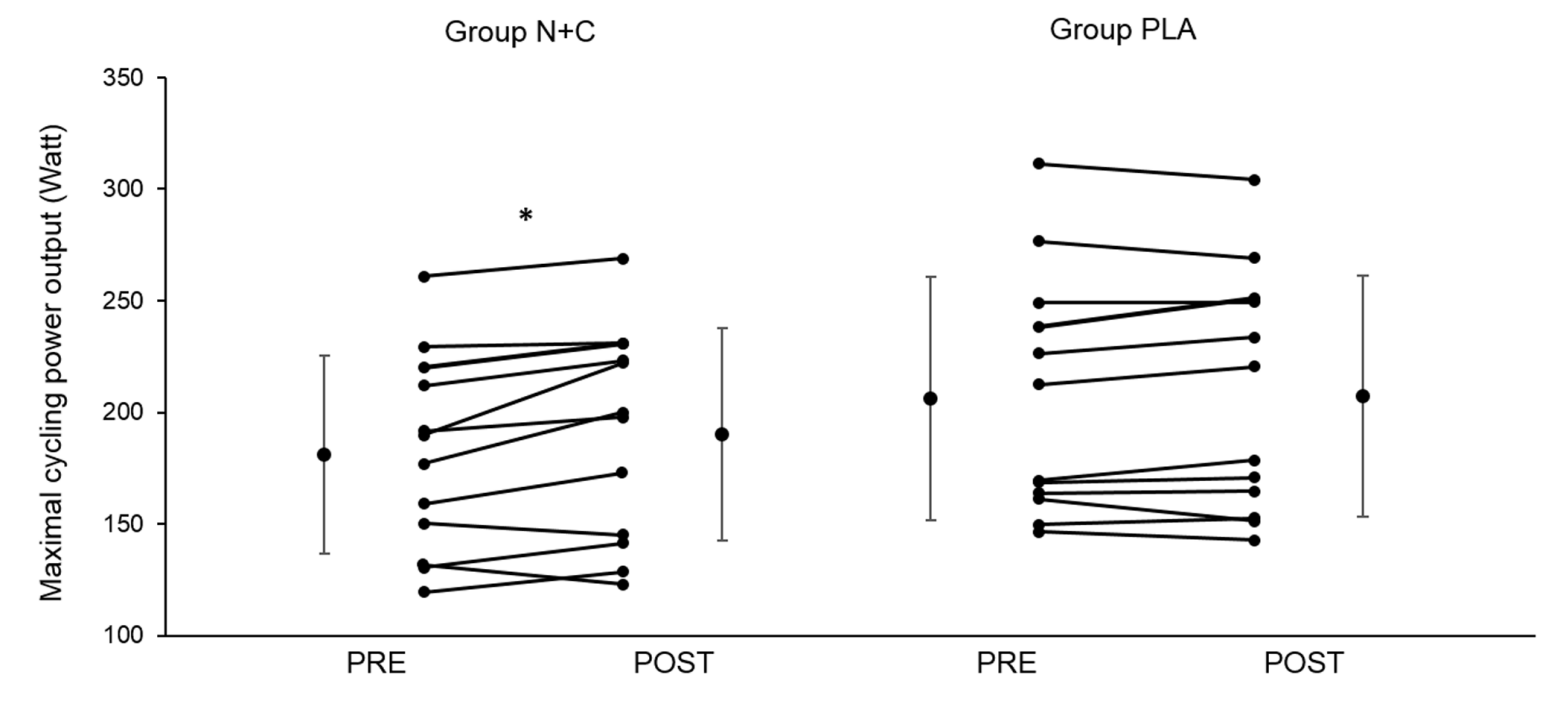
**Individual and group mean changes in maximal cycling power output before and after one month of placebo or nitrate and citrulline intake in older adults**. N+C, nitrate + citrulline; PLA, placebo; PRE, measure before the supplementation period; POST, measure after the supplementation period; * significant difference between PRE and POST; n=24.

There was no significant difference between groups for baseline maximal power output and VO_2_ (all p > 0.05). The increase in maximal power output between PRE and POST was significantly larger in the N+C group compared to PLA (p < 0.05, Table 5). Figure 2 shows heart rate and VO_2_ kinetics during the cycling exercise. There was a significant ANOVA main group effect on PRE-POST changes for heart rate and VO_2_ during cycling (at 25%, 50%, 75%, and 100% of the first test duration, i.e. at isowatt). The reduction in heart rate and VO_2_ was significantly larger in the N+C group compared to PLA. However, there was no effect on maximal heart rate and maximal VO_2_ (all p > 0.05; Figure 2, Table 5 and Figure 3) nor on submaximal and maximal minute ventilation (results not shown; all p > 0.05). There was no significant difference between groups for TSI (Table 4) and all other NIRS parameters (results not shown; all p > 0.05) during cycling.

## DISCUSSION

The main results of the present study are that, chronic NO precursor ingestion in healthy older adults i) decreased mean arterial BP but had no effect on arterial stiffness, post-ischemic vasodilation, and cardiovascular and cerebrovascular responses to hypercapnia and hypoxia, ii) had no effect on muscle and cerebral oxygenation during exercise, iii) had no effect on muscle strength and endurance during isometric knee extensions, and iv) increased maximal power output and decreased submaximal heart rate and VO_2_ during cycling. Taken together, our findings suggest that, in healthy older adults, a one-month supplementation of both NOS-independent and NOS-dependent pathways can improve arterial BP and increase maximal cycling capacity possibly due to a reduction in the O_2_ cost of cycling.

### Vascular function

Results from meta-analysis showed that in healthy subjects, acute and chronic (1 to 6 weeks) nitrate supplementation induces a mean SBP reduction of ∼4 mmHg [34,35]. In older adults, previous studies reported an improvement [23,36,37] or no change [38,39] in BP. Regarding citrulline, recent reviews reported that chronic intake has little or no effect on resting BP in healthy subjects [40] while in hypertensive patients it improves significantly endothelial dysfunction [22]. In the present study, 4 weeks of daily nitrate and citrulline intake induced a 7.5 and 6.8 mmHg reduction in SBP and MBP in older adults respectively, confirming the beneficial effect of NO precursor supplementation on resting BP. Based on the current knowledge regarding the impact of ageing on the endothelium function and NO metabolism [41], this beneficial effect could be due to both improved NOS-independent (i.e. enhanced nitrite reduction to NO) and/or NOS-dependant (i.e. increased NO production by NOS due to enhanced citrulline-arginine availability) NO production pathways.

This positive effect of NO precursors on BP was however not associated with an improvement in PWV and post-ischemic vasodilation. Arterial stiffness as assessed by PWV results from two distinct components in the arterial media: a structural and a dynamic component. The structural component is represented by the collagen and elastin fibers as well as other connecting molecules. The dynamic component is represented by the tone of smooth muscle cells, especially in the more muscular arteries, which is dependent on released of vasoactive substances such as NO [42]. Previous studies have shown an improvement [43,44] or no change [45,46] in PWV following chronic nitrate or citrulline intake in healthy subjects. Regional heterogeneity in arterial stiffening has been reported with advancing age [47,48]. There is a marked increase in aortic stiffness due to wall damage with ageing, while peripheral arterial stiffness is generally preserved in individuals >50 years. Since NO could lower vascular smooth muscle tone especially in more muscular arteries, NO precursor intake could reduce brachial BP without altering stiffness in larger elastic arteries, as observed in the present study.

Previous studies reported an improvement in post-ischemic vasodilation following both acute and chronic nitrate supplementation in patients [49], healthy adults [50] as well as in older adults [51]. Since the nitrate-nitrite-NO pathway contributes to NO production especially under hypoxic conditions [52], ischemic conditions may be particularly prone to show the positive effect of nitrate supplementation. A recent meta-analysis has shown that nitrate intake increases post-ischemic vasodilation to a greater extent in patients with impaired cardiovascular status compared with healthy subjects [53], suggesting that individuals with impaired endothelial function are more prone to benefit from nitrate intake. Conversely, improvement in endothelial function has not been reported following acute or chronic (~7 days) intake of citrulline in healthy subjects [4,33,54]. While a reduction in arterial oxygen pressure could lead to NOS-activity alteration, the previous and present results suggest that arginine bioavailability may not to be a limiting factor during post-ischemic vasodilation. One possible explanation for the lack of nitrate effect in the present study compared to previous studies mentioned above is that vascular dysfunction in older adults might be explained by other factor than NO bioavailability, e.g. by smooth muscle cell structure alteration [55]. While vascular alteration in patients is often characterised by low arginine/asymmetric dimethyl-arginine ratio, indicating a reduction of NO synthesis by NOS, in older adults this ratio is close to young healthy subject level [17]. Another aspect to consider is that post-ischemic vasodilatation has been assessed by Doppler ultrasound in the previous studies cited above while NIRS was used in the present study as an indirect, semi-quantitative measure of microvascular blood flow. We assessed microvascular function by NIRS since it may be more sensitive to the cardiovascular risk [56].

### Hypercapnic and hypoxic responses

NO has been shown to play a significant role in the regulation of blood flow under hypercapnic [57] and hypoxic [58] conditions. To our knowledge, this is the first study assessing the effect of NO precursor intake on hypercapnic cardiorespiratory and cerebral responses. The lack of effect of NO precursors on cerebrovascular response to hypercapnia may due to the multi-factorial regulation of CO_2_ responses, not only involving NO. For instance, cerebral autoregulation is known to rely on mechanisms involving adenosine, prostaglandins and anaerobic neuronal metabolism [59].

Since NOS-independent NO synthesis is facilitated by the presence of deoxyhaemoglobin [60,61], it has been postulated that an upregulation of the nitrate-nitrite-NO pathway could increase blood flow where O_2_ supply is limited [52]. There are limited and contrasted available data on the effect of NO precursor intake on hypoxic physiological responses in young healthy adults, and no study in older adults. While Shannon et al. [62] have shown that acute and chronic nitrate intake increased arterial oxygen saturation and cerebral but not muscle oxygenation during exercise at 4300 m, Masschelein et al. [63] have shown that chronic nitrate intake increased muscle but no cerebral oxygenation during exercise at 5000 m. In the present study, cerebral and muscle oxygenation measured by NIRS showed that both nitrate and citrulline chronic intake had no effect on hypoxic responses at rest as well as during submaximal exercise in healthy older individuals. Hence, despite the potential down regulation associated with hypoxia on NO production by the NOS dependent pathway, supplementing NOS with citrulline as well as the NOS-independent pathway with nitrate may not improve hypoxic responses. As recently emphasized in the review by Shannon et al. [64], further studies specifically focusing on the effect of NO precursors on hypoxic responses are however required.

### Incremental knee extension test

The unchanged knee extensor MVC following chronic NO precursor intake in older individuals is consistent with the literature regarding NO precursor effects on maximal force production in healthy adults [65–69]. In addition, the present study showed for the first time in older individuals no effect of NO precursors on isolated muscle endurance (i.e. total number of knee extensions), which is in contrast to previous results obtained in healthy young adults [65,67,68]. The lack of NO precursor effect on knee extension performance is consistent with the similar muscle and cerebral oxygenation measured by NIRS during exercise. Hence, in older adult, NO bioavailability may not be the limiting factor for muscle and cerebral oxygen delivery during isolated muscle exercise and as a consequence chronic NO precursor intake did not improve knee extension performance.

### Incremental cycling exercise

Previous studies in healthy subjects showed a positive effect of citrulline [70] or nitrate [71,72] supplementation on exercise endurance performance. An improvement in O_2_ cost during exercise has also been reported [73], that could be a result of an increase in mitochondrial function and oxidative phosphorylation efficiency [74]. Regarding older adults, four out of five studies assessing exercise performance found positive effects of chronic nitrate supplementation on time to exhaustion during submaximal exercise [23,24,37] and in VO_2_ response time [25], while only one study showed no significant effect on maximal exercise performances [26]. In the present study, 4 weeks of nitrate and citrulline supplementation reduced submaximal cycling exercise VO_2_ and heart rate. This effect was associated with a significant increase in maximal cycling power output of 5.2% in the N+C group. Taken together, these results suggest that chronic NO precursor intake reduces submaximal exercise heart rate and increases whole body exercise endurance performance by reducing the O_2_ cost of cycling. This might be due to an improvement in the ATP-O_2_ ratio and/or the ATP cost of muscle contraction following increased NO bioavailability [71]. These results suggest a potential interest of NO precursor supplementation to increase exercise tolerance and quality of life in patient, in particular in the context of physical rehabilitation [75]. The increase in cycling performance despite no difference in muscle and cerebral oxygenation patterns between groups suggests that this ergogenic effect of NO precursor intake in healthy older adults may not to be due to an improvement in muscle and cerebral perfusion and oxygen delivery.

### Methodological consideration

Blood concentrations of citrulline and nitrate were not assessed in the present study. Nevertheless, previous studies have reported significant increases in blood concentrations of NO metabolites or citrulline after similar nitrate or citrulline acute and chronic supplementations [4,76]. In contrast to previous studies assessing subjects on average 2 to 3 h after the last nitrate or citrulline intake, in the present study all tests were performed at least 6 h after the last NO precursor intake to avoid the acute effect of the supplementation and, instead, to focus on the chronic, long-lasting effect of the supplementation. Pharmacokinetics studies have shown that blood nitrite and arginine concentrations reach a peak 2 to 3 h after nitrate or citrulline supplementation before progressively returning to baseline values 5 to 8 h after intake [77,78], these kinetics remaining identical even after chronic supplementation [4,77,79]. Hence, in the present study, NO bioavailability during the post-supplementation testing session (at least 6 h after the last intake) may have been lower than in previous studies having assessed the effect of chronic NO precursor supplementation within 2-3 h after the last intake. This could account for the absence of some significant effects in the present work (e.g. on arterial stiffness and endothelial function) compared to previous studies. Conversely, the significant improvements in BP and cycling exercise responses observed in the N+C group indicate that chronic nitrate and citrulline supplementation in healthy older individuals induces positive outcomes due to mechanisms beyond those induced by acute NO precursor intake, *e.g.* a permanent increase in NO bioavailability and/or changes in muscle metabolic or contractile efficiency.

At last, this study included both males and females and did not detect significant sex difference for any parameter investigated. However, further studies with larger sample size are required to evaluate potential differences regarding the effects of NO precursors between females and males since female hormonal variations are known to affect vascular function and ageing [80].

## CONCLUSION

The present study shows that chronic nitrate and citrulline intake significantly decreased arterial BP, submaximal VO_2_ and heart rate during cycling exercise, and increased maximal cycling power output in healthy older adults. This was associated with no change in arterial stiffness, vascular reactivity, cerebral and muscle oxygenation during exercise and isolated knee extensor muscle strength and endurance. Hence, this study suggests that chronic supplementation of NOS-dependent and independent NO production pathways in older adults has positive effects on BP and whole body exercise performance which are important health-related physiological outcomes especially regarding ageing and cardiovascular risks.

## MATERIALS AND METHODS

### Subjects

Twenty-four subjects (12 males, age 64 ± 2 years; body mass 73.5 ± 6.1 kg; height 178 ± 5 cm; 12 females, age 62 ± 2 years; body mass 57.1 ± 4.2 kg; height 154 ± 4 cm) were enrolled according to the following inclusion criteria: healthy, no more than 2 sessions of physical activity at low to moderate intensity per week, age between 60 and 70 years old, body mass index between 18 and 30 kg·m^-2^, non-smokers and no medication (except hormonal treatment). All participants had to be free from any use of food supplements or particular diet. The study was approved by the local ethics committee (CPP Sud-Est V, 2014-A01876-41) and performed according to the Declaration of Helsinki. Subjects were fully informed of the procedure and risks involved and gave their written consent.

### Study design

**Figure 4:**
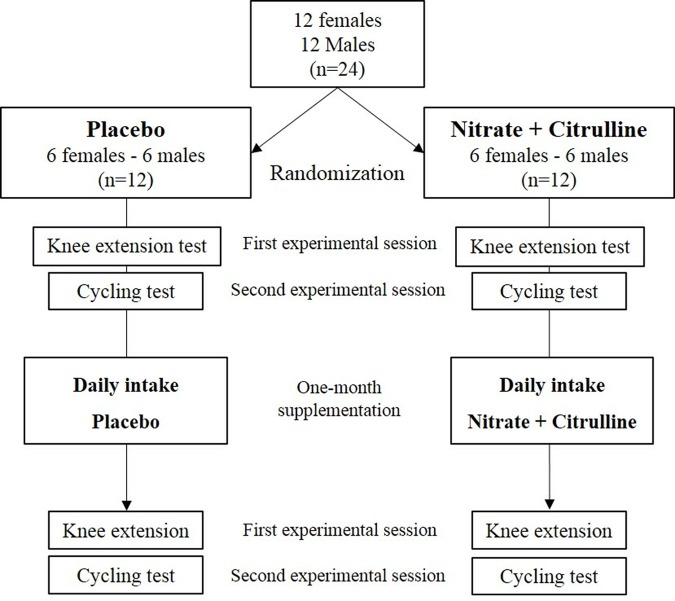
Overview of the study design.

In this double blind, randomized study, after a familiarization session, each participant was tested before (2 experimental sessions) and after (2 experimental sessions) one month of daily NO precursors or placebo intake (Figure 4). A recovery period of at least two days separated each experimental session. All tests were performed at least 6 h after the last supplementation to avoid the acute effect of NO precursors. The day before each testing session and on the testing days, subjects were instructed to adhere to their normal living and dietary routines, to avoid caffeine, dehydration or excessive hydration. During the supplementation period, subjects were also instructed to refrain from using any kind of mouth wash. Nutrition and physical activity before and during the protocol were recorded on a diary and controlled by the investigators.

During the first experimental session, resting arterial BP (Digital Blood Pressure Monitor, A&D Medical, Sydney, Australia) and arterial stiffness as pulse wave velocity (PWV; COMPLIOR device, ALAM Medical, Colson, Les Lilas, France) were measured three times. Then, subjects performed an ischemia-reperfusion test on the lower limb to evaluate the NO dependent vasodilation (see below). Following this evaluation, participants sat in a custom-built chair, with the right knee flexed at 90º and the ankle fixed to a strain gauge (Meiri F2732, Celians, Montauban, France), and performed an incremental intermittent isometric knee extension test. After a standardized 5-min warm up phase, subjects performed four maximal voluntary contractions (MVC) with 1 min of rest in between and MVC was determined as the highest force peak among the four trials. Then, exercise started at a target force of 35% MVC which was increased by 5% every 4 min. Subjects followed the instructions of a soundtrack to contract (5 s) and relax (4 s) the right quadriceps according to a visual signal displaying the target force level. Task failure was defined as the inability of the subject to perform three consecutive contractions adequately (i.e. if the contraction was not of 5-s duration or if the mean contraction torque was lower than the target torque for >2s). During the knee extension test, cerebral and muscle oxygenation was measured by near infrared spectroscopy (NIRS) (see below).

During the second experimental session, subjects breathed through a face-mask and were blinded to the inhaled gas mixture composition delivered by an IsoCap-Altitrainer 200® (SMTEC, Nyon, Switzerland). During the initial phase, subjects sat quietly in a semi-recumbent position and inhaled various gas mixtures in order to evaluate the cerebrovascular reactivity: first, subjects inhaled a normoxic gas mixture for 5 min (inspiratory oxygen fraction, FiO_2_ = 0.21; normoxia); then, they inhaled a normoxic hypercapnic gas mixture (FiCO_2_ = 0.04-0.07%) individually and continuously adjusted to induce for 5 min an end-tidal partial pressure of CO_2_ (PetCO_2_) 10 mmHg above the initial normoxic PetCO_2_; after another 5-min normoxic phase, subjects inhaled a hypoxic gas mixture (FiO_2_ = 0.12) for 5 min. After these resting measurements, subjects continued to inhale the hypoxic gas mixture (FiO_2_ = 0.12) and were installed on a cycle ergometer (Lode® CORIVAL, Groningen, The Netherlands) to start cycling at a constant-load of 70 W (males) or 50 W (females) for 10 min (hypoxic cycling), followed by another 10-min constant cycling phase at the same workload while inhaling a normoxic gas mixture (normoxic cycling). These two 10-min cycling phases allowed evaluating the cardiovascular exercise hypoxic responses [81]. Finally, starting from this initial workload, a maximal incremental cycling test was performed with an increment of 10 W every minute until volitional exhaustion. Throughout the test, cerebral and muscle oxygenation was measured by NIRS, arterial oxygen saturation was recorded by finger pulse oximetry (Masimo Radical 7, Masimo Corp., Irvine, CA), BP was measured on the right arm with a digital pressure monitor system (Digital Blood Pressure Monitor A&D Medical, Sydney, Australia) and minute ventilation (VE) and gas exchanges (VO_2_, VCO_2_, PETCO_2_) were monitored breath-by-breath using a metabolic cart (MetaMax 3B, Cortex Biophysik GmbH, Leipzig, Germany). Gas analysers and volume transducers were calibrated prior to each test with a 3-L syringe and references gases, respectively, according to manufacturer's instructions.

These two experimental sessions (PRE) were repeated identically after the one-month supplementation period (POST).

### NO precursor supplementation

The NO precursor beverage (N+C) was composed of nitrate-rich natural dried salad extract and citrulline. It supplied 520 mg of nitrate (8.6 mmol) and 6 g of citrulline. These doses have been shown to respectively increase significantly plasma nitrite and arginine without causing any intestinal problems. The placebo beverage (PLA) was composed of nitrate-free cherry tail juice and was of similar colour and taste than the N+C beverage. The two supplements had the same carbohydrate profile. Both the experimenters and the subjects were blinded for the composition of the beverages. After the first initial evaluation visits, subjects were randomized into the N+C or the PLA groups and were asked to drink the beverage every morning.

### Pulse wave velocity

PWV was analysed with a non-invasive automatic device (COMPLIOR device, ALAM Medical). Arterial stiffness measured by PWV has been shown to be acutely influenced by vascular tone and constitutively released NO [82]. The PWV measurement technique has been described previously elsewhere [83,84]. Briefly, common carotid artery and femoral artery pressure waveforms were recorded noninvasively. The pressure waveforms were digitized at the sample acquisition frequency of 500 Hz. A pre-processing system automatically analysed the gain in each waveform and adjusted it for equality of the 2 signals. When the operator observed a pulse waveform of sufficient quality on the computer screen, digitization was suspended and calculation of the time delay between the 2 pressure upstrokes was initiated. Measurements were repeated over at least 5 different cardiac cycles, and the mean was used for the final analysis. The distance travelled by the pulse wave was measured over the body surface as the distance between the 2 recording sites (D), whereas pulse transit time (t) was automatically determined by the Complior device; PWV was automatically calculated as PWV=D/t, and 80% of this distance defined the pulse wave travelled distance (common carotid artery-common femoral artery × 0.8).

### Ischemia-reperfusion test

A pneumatic cuff (Santelec, Cestas, France) was positioned proximally on the right thigh. After completion of a 5-min baseline phase, a rapid arterial occlusion (<30 s) of the right leg was induced by manual inflation of the pneumatic cuff at 250 mmHg. The cuff remained inflated for 5 min. The arterial cuff was rapidly deflated in less than 5 sec to initiate the reperfusion phase. The reperfusion was monitored for 5 min. During the reperfusion phase, kinetics of NIRS signals were recorded to evaluate post-ischemic vasodilation as previously described [85].

### Near infrared spectroscopy (NIRS)

Oxy[HbO_2_]-, deoxy[HHb]-, total[HbTot]-haemoglobin concentration and tissue saturation index (TSI) changes were estimated throughout testing sessions over multiple sites using a two-wavelength (780 and 850 nm) multichannel, continuous wave NIRS system (Oxymon MkIII, Artinis Medical Systems, the Netherlands). Quadriceps muscle hemodynamic was assessed from the right *vastus lateralis* using a 4-cm interoptodes distance. Probe holder was secured to the skin using double-sided tape and covered with a black sweatband to shield the optodes from ambient light. Left pre-frontal cortex hemodynamic was assessed between Fp1 and F3 locations according to the international 10–20 EEG system with 3.5-cm interoptodes distance. The probe holders were secured to the skin with double-sided tape and maintained with Velcro headbands. Data were recorded continuously at 10 Hz and filtered with a 1-s width moving Gaussian smoothing algorithm before analysis.

### Data analysis

Ischemia-reperfusion NIRS response was characterized by changes in HbTot concentration as a quantitative index of blood volume and by changes in HbO_2_ concentrations as a qualitative index of tissue oxygen delivery during the post-ischemia phase. During the reperfusion phase, the difference between the value at the end of the ischemia phase and the maximal value reached during the reperfusion phase (∆max/min) represents the lower-limb post-ischemic vascular reactivity. Resting hypercapnic and hypoxic responses were characterized by changes in cardiovascular and cerebrovascular parameters between the initial 5-min normoxic phase and the 5-min hypoxic or 5-min hypercapnic phases (the last 60 s of each phase were used for analysis). Exercise hypoxic responses were characterized by changes in cardiovascular and cerebrovascular parameters between the 10-min normoxic cycling phase and the 10-min hypoxic cycling phase (the last 60 s of each phase were used for analysis).

Owing to the between-subject variability in time to task failure during knee extension exercise and incremental cycling test, all data were normalized as a percentage of endurance time [86]. Data from experimental sessions before and after the supplementation period were compared at different time points: i) at 25% (25%), ii) at 50% (50%), iii) 75% (75%), iv) 100% (100%) of the duration of the test performed before the supplementation period, and iv) during the last 30 s of the knee extension exercise or incremental cycling test (exhaustion).

### Statistical analysis

Power assessment for the primary outcome (exercise performance) was based on a minimum expected NO precursor effect of 10%. With an α level of 5% and power of 80%, 24 subjects were required. Statistical analysis was conducted with n = 24 for all evaluations except for TSI in pre-frontal cortex and quadriceps muscles (n = 23) due to technical issues. Data were analyzed with SPSS v.24 software (SPSS Inc, Chicago, United states). Data from PRE and POST supplementation period in each group were compared using two-way (N+C/PLA group × PRE/POST session) ANOVA after establishing that data conformed to a normal distribution (Shapiro-Wilk test) and homogeneity of variance (Levene’s test). Least Squares Difference (LSD) post hoc analyses were performed when a significant ANOVA effect was identified. Partial eta square (pη2) values are reported as measures of effect size, with moderate and large effects considered for pη2 ≥ 0.07 and pη2 ≥ 0.14, respectively (Cohen, 1988). Data were also analyzed as differences between PRE and POST supplementation period (PRE-POST changes). In this case, PRE-POST changes between the N+C and PLA groups were compared with unpaired t-test and Cohen’s delta (d) determined the effect size and practical significance of N+C effect. Effect sizes were classified as small if d ≤ 0.2, medium if d ≈ 0.5, and large if d ≥ 0.8 [87]. For all statistical analyses, a two-tailed alpha level of 0.05 was used as the cut off for significance. All data are presented as mean values ± SD.

## References

[1] Hirst DG, Robson T. Nitric oxide physiology and pathology. Methods Mol Biol. 2011; 11 10 3315 3332:1–13. 10.1007/978-1-61737-964-2_1 21161625

[2] Chin-Dusting JP, Willems L, Kaye DM. L-arginine transporters in cardiovascular disease: a novel therapeutic target. Pharmacol Ther. 2007; 116:428–36. 10.1016/j.pharmthera.2007.08.001 17915331

[3] Lundberg JO, Govoni M. Inorganic nitrate is a possible source for systemic generation of nitric oxide. Free Radic Biol Med. 2004; 37:395–400. 10.1016/j.freeradbiomed.2004.04.027 15223073

[4] Schwedhelm E, Maas R, Freese R, Jung D, Lukacs Z, Jambrecina A, Spickler W, Schulze F, Böger RH. Pharmacokinetic and pharmacodynamic properties of oral L-citrulline and L-arginine: impact on nitric oxide metabolism. Br J Clin Pharmacol. 2008; 65:51–59. 10.1111/j.1365-2125.2007.02990.x 17662090 PMC2291275

[5] Waugh WH, Daeschner CW 3rd, Files BA, McConnell ME, Strandjord SE. Oral citrulline as arginine precursor may be beneficial in sickle cell disease: early phase two results. J Natl Med Assoc. 2001; 93:363–71. 11688916 PMC2594068

[6] Wijnands KA, Vink H, Briedé JJ, van Faassen EE, Lamers WH, Buurman WA, Poeze M. Citrulline a more suitable substrate than arginine to restore NO production and the microcirculation during endotoxemia. PLoS One. 2012; 7:e37439. 10.1371/journal.pone.0037439 22666356 PMC3362574

[7] Gilligan DM, Panza JA, Kilcoyne CM, Waclawiw MA, Casino PR, Quyyumi AA. Contribution of endothelium-derived nitric oxide to exercise-induced vasodilation. Circulation. 1994; 90:2853–58. 10.1161/01.CIR.90.6.2853 7994830

[8] Casey DP, Walker BG, Ranadive SM, Taylor JL, Joyner MJ. Contribution of nitric oxide in the contraction-induced rapid vasodilation in young and older adults. J Appl Physiol (1985). 2013; 115:446–55. 10.1152/japplphysiol.00446.2013 23788575 PMC3742946

[9] Dawson TM, Snyder SH. Gases as biological messengers: nitric oxide and carbon monoxide in the brain. J Neurosci. 1994; 14:5147–59. 10.1523/JNEUROSCI.14-09-05147.1994 8083727 PMC6577075

[10] White RP, Vallance P, Markus HS. Effect of inhibition of nitric oxide synthase on dynamic cerebral autoregulation in humans. Clin Sci (Lond). 2000; 99:555–60. 10.1042/cs0990555 11099400

[11] Garry PS, Ezra M, Rowland MJ, Westbrook J, Pattinson KT. The role of the nitric oxide pathway in brain injury and its treatment--from bench to bedside. Exp Neurol. 2015; 263:235–43. 10.1016/j.expneurol.2014.10.017 25447937

[12] Attwell D, Buchan AM, Charpak S, Lauritzen M, Macvicar BA, Newman EA. Glial and neuronal control of brain blood flow. Nature. 2010; 468:232–43. 10.1038/nature09613 21068832 PMC3206737

[13] Versari D, Daghini E, Virdis A, Ghiadoni L, Taddei S. Endothelial dysfunction as a target for prevention of cardiovascular disease. Diabetes Care. 2009 (Suppl 2); 32:S314–21. 10.2337/dc09-S330 19875572 PMC2811443

[14] Brandes RP, Fleming I, Busse R. Endothelial aging. Cardiovasc Res. 2005; 66:286–94. 10.1016/j.cardiores.2004.12.027 15820197

[15] Forte P, Copland M, Smith LM, Milne E, Sutherland J, Benjamin N. Basal nitric oxide synthesis in essential hypertension. Lancet. 1997; 349:837–42. 10.1016/S0140-6736(96)07631-3 9121259

[16] Halcox JP, Schenke WH, Zalos G, Mincemoyer R, Prasad A, Waclawiw MA, Nour KR, Quyyumi AA. Prognostic value of coronary vascular endothelial dysfunction. Circulation. 2002; 106:653–58. 10.1161/01.CIR.0000025404.78001.D8 12163423

[17] Gates PE, Boucher ML, Silver AE, Monahan KD, Seals DR. Impaired flow-mediated dilation with age is not explained by L-arginine bioavailability or endothelial asymmetric dimethylarginine protein expression. J Appl Physiol (1985). 2007; 102:63–71. 10.1152/japplphysiol.00660.2006 16946027

[18] Förstermann U, Münzel T. Endothelial nitric oxide synthase in vascular disease: from marvel to menace. Circulation. 2006; 113:1708–14. 10.1161/CIRCULATIONAHA.105.602532 16585403

[19] Williams PT. Physical fitness and activity as separate heart disease risk factors: a meta-analysis. Med Sci Sports Exerc. 2001; 33:754–61. 10.1097/00005768-200105000-00012 11323544 PMC2821586

[20] Gray BJ, Stephens JW, Williams SP, Davies CA, Turner D, Bracken RM, and Prosiect Sir Gâr Group. Cardiorespiratory fitness is a stronger indicator of cardiometabolic risk factors and risk prediction than self-reported physical activity levels. Diab Vasc Dis Res. 2015; 12:428–35. 10.1177/1479164115599907 26361778

[21] Stanaway L, Rutherfurd-Markwick K, Page R, Ali A. Performance and Health Benefits of Dietary Nitrate Supplementation in Older Adults: A Systematic Review. Nutrients. 2017; 9:9. 10.3390/nu9111171 29077028 PMC5707643

[22] Allerton TD, Proctor DN, Stephens JM, Dugas TR, Spielmann G, Irving BA. l-Citrulline Supplementation: Impact on Cardiometabolic Health. Nutrients. 2018; 10:10. 10.3390/nu10070921 30029482 PMC6073798

[23] Berry MJ, Justus NW, Hauser JI, Case AH, Helms CC, Basu S, Rogers Z, Lewis MT, Miller GD. Dietary nitrate supplementation improves exercise performance and decreases blood pressure in COPD patients. Nitric Oxide. 2015; 48:22–30. 10.1016/j.niox.2014.10.007 25445634 PMC4411191

[24] Eggebeen J, Kim-Shapiro DB, Haykowsky M, Morgan TM, Basu S, Brubaker P, Rejeski J, Kitzman DW. One Week of Daily Dosing With Beetroot Juice Improves Submaximal Endurance and Blood Pressure in Older Patients With Heart Failure and Preserved Ejection Fraction. JACC Heart Fail. 2016; 4:428–37. 10.1016/j.jchf.2015.12.013 26874390 PMC4892939

[25] Kelly J, Fulford J, Vanhatalo A, Blackwell JR, French O, Bailey SJ, Gilchrist M, Winyard PG, Jones AM. Effects of short-term dietary nitrate supplementation on blood pressure, O2 uptake kinetics, and muscle and cognitive function in older adults. Am J Physiol Regul Integr Comp Physiol. 2013; 304:R73–83. 10.1152/ajpregu.00406.2012 23174856

[26] Siervo M, Oggioni C, Jakovljevic DG, Trenell M, Mathers JC, Houghton D, Celis-Morales C, Ashor AW, Ruddock A, Ranchordas M, Klonizakis M, Williams EA. Dietary nitrate does not affect physical activity or outcomes in healthy older adults in a randomized, cross-over trial. Nutr Res. 2016; 36:1361–69. 10.1016/j.nutres.2016.11.004 27890482

[27] Larsen FJ, Ekblom B, Sahlin K, Lundberg JO, Weitzberg E. Effects of dietary nitrate on blood pressure in healthy volunteers. N Engl J Med. 2006; 355:2792–93. 10.1056/NEJMc062800 17192551

[28] Kapil V, Milsom AB, Okorie M, Maleki-Toyserkani S, Akram F, Rehman F, Arghandawi S, Pearl V, Benjamin N, Loukogeorgakis S, Macallister R, Hobbs AJ, Webb AJ, Ahluwalia A. Inorganic nitrate supplementation lowers blood pressure in humans: role for nitrite-derived NO. Hypertension. 2010; 56:274–81. 10.1161/HYPERTENSIONAHA.110.153536 20585108

[29] Presley TD, Morgan AR, Bechtold E, Clodfelter W, Dove RW, Jennings JM, Kraft RA, King SB, Laurienti PJ, Rejeski WJ, Burdette JH, Kim-Shapiro DB, Miller GD. Acute effect of a high nitrate diet on brain perfusion in older adults. Nitric Oxide. 2011; 24:34–42. 10.1016/j.niox.2010.10.002 20951824 PMC3018552

[30] Webb AJ, Patel N, Loukogeorgakis S, Okorie M, Aboud Z, Misra S, Rashid R, Miall P, Deanfield J, Benjamin N, MacAllister R, Hobbs AJ, Ahluwalia A. Acute blood pressure lowering, vasoprotective, and antiplatelet properties of dietary nitrate via bioconversion to nitrite. Hypertension. 2008; 51:784–90. 10.1161/HYPERTENSIONAHA.107.103523 18250365 PMC2839282

[31] Miller GD, Marsh AP, Dove RW, Beavers D, Presley T, Helms C, Bechtold E, King SB, Kim-Shapiro D. Plasma nitrate and nitrite are increased by a high-nitrate supplement but not by high-nitrate foods in older adults. Nutr Res. 2012; 32:160–68. 10.1016/j.nutres.2012.02.002 22464802 PMC3319660

[32] Gonzales JU, Raymond A, Ashley J, Kim Y. Does l-citrulline supplementation improve exercise blood flow in older adults? Exp Physiol. 2017; 102:1661–71. 10.1113/EP086587 28940638 PMC5999519

[33] Kim IY, Schutzler SE, Schrader A, Spencer HJ, Azhar G, Deutz NE, Wolfe RR. Acute ingestion of citrulline stimulates nitric oxide synthesis but does not increase blood flow in healthy young and older adults with heart failure. Am J Physiol Endocrinol Metab. 2015; 309:E915–24. 10.1152/ajpendo.00339.2015 26442881 PMC4669336

[34] Siervo M, Lara J, Ogbonmwan I, Mathers JC. Inorganic nitrate and beetroot juice supplementation reduces blood pressure in adults: a systematic review and meta-analysis. J Nutr. 2013; 143:818–26. 10.3945/jn.112.170233 23596162

[35] Ashor AW, Lara J, Siervo M. Medium-term effects of dietary nitrate supplementation on systolic and diastolic blood pressure in adults: a systematic review and meta-analysis. J Hypertens. 2017; 35:1353–59. 10.1097/HJH.0000000000001305 28319596

[36] Kemmner S, Lorenz G, Wobst J, Kessler T, Wen M, Günthner R, Stock K, Heemann U, Burkhardt K, Baumann M, Schmaderer C. Dietary nitrate load lowers blood pressure and renal resistive index in patients with chronic kidney disease: A pilot study. Nitric Oxide. 2017; 64:7–15. 10.1016/j.niox.2017.01.011 28137609

[37] Kenjale AA, Ham KL, Stabler T, Robbins JL, Johnson JL, Vanbruggen M, Privette G, Yim E, Kraus WE, Allen JD. Dietary nitrate supplementation enhances exercise performance in peripheral arterial disease. J Appl Physiol (1985). 2011; 110:1582–91. 10.1152/japplphysiol.00071.2011 21454745 PMC3119136

[38] Gilchrist M, Winyard PG, Aizawa K, Anning C, Shore A, Benjamin N. Effect of dietary nitrate on blood pressure, endothelial function, and insulin sensitivity in type 2 diabetes. Free Radic Biol Med. 2013; 60:89–97. 10.1016/j.freeradbiomed.2013.01.024 23395779

[39] Shepherd AI, Wilkerson DP, Fulford J, Winyard PG, Benjamin N, Shore AC, Gilchrist M. Effect of nitrate supplementation on hepatic blood flow and glucose homeostasis: a double-blind, placebo-controlled, randomized control trial. Am J Physiol Gastrointest Liver Physiol. 2016; 311:G356–64. 10.1152/ajpgi.00203.2016 27418682 PMC5076007

[40] Mirenayat MS, Moradi S, Mohammadi H, Rouhani MH. Effect of L-Citrulline Supplementation on Blood Pressure: a Systematic Review and Meta-Analysis of Clinical Trials. Curr Hypertens Rep. 2018; 20:98. 10.1007/s11906-018-0898-3 30284051

[41] Widlansky ME, Gokce N, Keaney JF Jr, Vita JA. The clinical implications of endothelial dysfunction. J Am Coll Cardiol. 2003; 42:1149–60. 10.1016/S0735-1097(03)00994-X 14522472

[42] Mirea O, Donoiu I, Pleşea IE. Arterial aging: a brief review. Rom J Morphol Embryol. 2012; 53:473–77. 22990535

[43] Bahra M, Kapil V, Pearl V, Ghosh S, Ahluwalia A. Inorganic nitrate ingestion improves vascular compliance but does not alter flow-mediated dilatation in healthy volunteers. Nitric Oxide. 2012; 26:197–202. 10.1016/j.niox.2012.01.004 22285857 PMC3405527

[44] Ochiai M, Hayashi T, Morita M, Ina K, Maeda M, Watanabe F, Morishita K. Short-term effects of L-citrulline supplementation on arterial stiffness in middle-aged men. Int J Cardiol. 2012; 155:257–61. 10.1016/j.ijcard.2010.10.004 21067832

[45] Lara J, Ogbonmwan I, Oggioni C, Zheng D, Qadir O, Ashor A, Brandt K, Mathers JC, Siervo M. Effects of handgrip exercise or inorganic nitrate supplementation on 24-h ambulatory blood pressure and peripheral arterial function in overweight and obese middle age and older adults: A pilot RCT. Maturitas. 2015; 82:228–35. 10.1016/j.maturitas.2015.07.028 26316026

[46] Bondonno CP, Liu AH, Croft KD, Ward NC, Yang X, Considine MJ, Puddey IB, Woodman RJ, Hodgson JM. Short-term effects of nitrate-rich green leafy vegetables on blood pressure and arterial stiffness in individuals with high-normal blood pressure. Free Radic Biol Med. 2014; 77:353–62. 10.1016/j.freeradbiomed.2014.09.021 25261227

[47] Mitchell GF, Parise H, Benjamin EJ, Larson MG, Keyes MJ, Vita JA, Vasan RS, Levy D. Changes in Arterial Stiffness and Wave Reflection With Advancing Age in Healthy Men and Women. Hypertension. 2004; 43:1239–45. 10.1161/01.HYP.0000128420.01881.aa 15123572

[48] Benetos A, Laurent S, Hoeks AP, Boutouyrie PH, Safar ME. Arterial alterations with aging and high blood pressure. A noninvasive study of carotid and femoral arteries. Arterioscler Thromb. 1993; 13:90–97. 10.1161/01.ATV.13.1.90 8422344

[49] Velmurugan S, Gan JM, Rathod KS, Khambata RS, Ghosh SM, Hartley A, Van Eijl S, Sagi-Kiss V, Chowdhury TA, Curtis M, Kuhnle GG, Wade WG, Ahluwalia A. Dietary nitrate improves vascular function in patients with hypercholesterolemia: a randomized, double-blind, placebo-controlled study. Am J Clin Nutr. 2016; 103:25–38. 10.3945/ajcn.115.116244 26607938 PMC4691670

[50] Heiss C, Meyer C, Totzeck M, Hendgen-Cotta UB, Heinen Y, Luedike P, Keymel S, Ayoub N, Lundberg JO, Weitzberg E, Kelm M, Rassaf T. Dietary inorganic nitrate mobilizes circulating angiogenic cells. Free Radic Biol Med. 2012; 52:1767–72. 10.1016/j.freeradbiomed.2012.02.051 22406434

[51] Rammos C, Hendgen-Cotta UB, Sobierajski J, Bernard A, Kelm M, Rassaf T. Dietary nitrate reverses vascular dysfunction in older adults with moderately increased cardiovascular risk. J Am Coll Cardiol. 2014; 63:1584–85. 10.1016/j.jacc.2013.08.691 23994403

[52] Lundberg JO, Weitzberg E, Gladwin MT. The nitrate-nitrite-nitric oxide pathway in physiology and therapeutics. Nat Rev Drug Discov. 2008; 7:156–67. 10.1038/nrd2466 18167491

[53] Jackson JK, Patterson AJ, MacDonald-Wicks LK, Oldmeadow C, McEvoy MA. The role of inorganic nitrate and nitrite in cardiovascular disease risk factors: a systematic review and meta-analysis of human evidence. Nutr Rev. 2018; 76:348–71. 10.1093/nutrit/nuy005 29506204

[54] Churchward-Venne TA, Cotie LM, MacDonald MJ, Mitchell CJ, Prior T, Baker SK, Phillips SM. Citrulline does not enhance blood flow, microvascular circulation, or myofibrillar protein synthesis in elderly men at rest or following exercise. Am J Physiol Endocrinol Metab. 2014; 307:E71–83. 10.1152/ajpendo.00096.2014 24824653

[55] Sehgel NL, Vatner SF, Meininger GA. “Smooth Muscle Cell Stiffness Syndrome”—Revisiting the Structural Basis of Arterial Stiffness. Front Physiol. 2015; 6:335. 10.3389/fphys.2015.00335 26635621 PMC4649054

[56] Gayda M, Juneau M, Tardif JC, Harel F, Levesque S, Nigam A. Cardiometabolic and traditional cardiovascular risk factors and their potential impact on macrovascular and microvascular function: preliminary data. Clin Hemorheol Microcirc. 2015; 59:53–65. 10.3233/CH-141816 24518371

[57] Iadecola C. Regulation of the cerebral microcirculation during neural activity: is nitric oxide the missing link? Trends Neurosci. 1993; 16:206–14. 10.1016/0166-2236(93)90156-G 7688160

[58] Singel DJ, Stamler JS. Chemical physiology of blood flow regulation by red blood cells: the role of nitric oxide and S-nitrosohemoglobin. Annu Rev Physiol. 2005; 67:99–145. 10.1146/annurev.physiol.67.060603.090918 15709954

[59] Willie CK, Tzeng YC, Fisher JA, Ainslie PN. Integrative regulation of human brain blood flow. J Physiol. 2014; 592:841–59. 10.1113/jphysiol.2013.268953 24396059 PMC3948549

[60] Cosby K, Partovi KS, Crawford JH, Patel RP, Reiter CD, Martyr S, Yang BK, Waclawiw MA, Zalos G, Xu X, Huang KT, Shields H, Kim-Shapiro DB, et al. Nitrite reduction to nitric oxide by deoxyhemoglobin vasodilates the human circulation. Nat Med. 2003; 9:1498–505. 10.1038/nm954 14595407

[61] Brooks J. The action of nitrite on haemoglobin in the absence of oxygen. Proc R Soc Lond B Biol Sci. 1937; 123:368–82. 10.1098/rspb.1937.0057

[62] Shannon OM, Duckworth L, Barlow MJ, Deighton K, Matu J, Williams EL, Woods D, Xie L, Stephan BC, Siervo M, O’Hara JP. Effects of Dietary Nitrate Supplementation on Physiological Responses, Cognitive Function, and Exercise Performance at Moderate and Very-High Simulated Altitude. Front Physiol. 2017; 8:401. 10.3389/fphys.2017.00401 28649204 PMC5465306

[63] Masschelein E, Van Thienen R, Wang X, Van Schepdael A, Thomis M, Hespel P. Dietary nitrate improves muscle but not cerebral oxygenation status during exercise in hypoxia. J Appl Physiol (1985). 2012; 113:736–45. 10.1152/japplphysiol.01253.2011 22773768

[64] Shannon OM, McGawley K, Nybäck L, Duckworth L, Barlow MJ, Woods D, Siervo M, O’Hara JP. “Beet-ing” the Mountain: A Review of the Physiological and Performance Effects of Dietary Nitrate Supplementation at Simulated and Terrestrial Altitude. Sports Med. 2017; 47:2155–69. 10.1007/s40279-017-0744-9 28577258 PMC5633647

[65] Porcelli S, Pugliese L, Rejc E, Pavei G, Bonato M, Montorsi M, La Torre A, Rasica L, Marzorati M. Effects of a Short-Term High-Nitrate Diet on Exercise Performance. Nutrients. 2016; 8. 10.3390/nu8090534 27589795 PMC5037521

[66] Hoon MW, Fornusek C, Chapman PG, Johnson NA. The effect of nitrate supplementation on muscle contraction in healthy adults. Eur J Sport Sci. 2015; 15:712–19. 10.1080/17461391.2015.1053418 26681629

[67] Haider G, Folland JP. Nitrate supplementation enhances the contractile properties of human skeletal muscle. Med Sci Sports Exerc. 2014; 46:2234–43. 10.1249/MSS.0000000000000351 24681572

[68] Fulford J, Winyard PG, Vanhatalo A, Bailey SJ, Blackwell JR, Jones AM. Influence of dietary nitrate supplementation on human skeletal muscle metabolism and force production during maximum voluntary contractions. Pflugers Arch. 2013; 465:517–28. 10.1007/s00424-013-1220-5 23354414

[69] Meirelles CM, Matsuura C. Acute supplementation of L-arginine affects neither strength performance nor nitric oxide production. J Sports Med Phys Fitness. 2018; 58:216–20. 27623757 10.23736/S0022-4707.16.06680-9

[70] Figueroa A, Wong A, Jaime SJ, Gonzales JU. Influence of L-citrulline and watermelon supplementation on vascular function and exercise performance. Curr Opin Clin Nutr Metab Care. 2017; 20:92–98. 10.1097/MCO.0000000000000340 27749691

[71] Jones AM, Thompson C, Wylie LJ, Vanhatalo A. Dietary Nitrate and Physical Performance. Annu Rev Nutr. 2018; 38:303–28. 10.1146/annurev-nutr-082117-051622 30130468

[72] McMahon NF, Leveritt MD, Pavey TG. The Effect of Dietary Nitrate Supplementation on Endurance Exercise Performance in Healthy Adults: A Systematic Review and Meta-Analysis. Sports Med. 2017; 47:735–56. 10.1007/s40279-016-0617-7 27600147

[73] Larsen FJ, Weitzberg E, Lundberg JO, Ekblom B. Effects of dietary nitrate on oxygen cost during exercise. Acta Physiol (Oxf). 2007; 191:59–66. 10.1111/j.1748-1716.2007.01713.x 17635415

[74] Larsen FJ, Schiffer TA, Borniquel S, Sahlin K, Ekblom B, Lundberg JO, Weitzberg E. Dietary inorganic nitrate improves mitochondrial efficiency in humans. Cell Metab. 2011; 13:149–59. 10.1016/j.cmet.2011.01.004 21284982

[75] Hambrecht R, Hilbrich L, Erbs S, Gielen S, Fiehn E, Schoene N, Schuler G. Correction of endothelial dysfunction in chronic heart failure: additional effects of exercise training and oral L-arginine supplementation. J Am Coll Cardiol. 2000; 35:706–13. 10.1016/S0735-1097(99)00602-6 10716474

[76] Wylie LJ, Kelly J, Bailey SJ, Blackwell JR, Skiba PF, Winyard PG, Jeukendrup AE, Vanhatalo A, Jones AM. Beetroot juice and exercise: pharmacodynamic and dose-response relationships. J Appl Physiol (1985). 2013; 115:325–36. 10.1152/japplphysiol.00372.2013 23640589

[77] Moinard C, Maccario J, Walrand S, Lasserre V, Marc J, Boirie Y, Cynober L. Arginine behaviour after arginine or citrulline administration in older subjects. Br J Nutr. 2016; 115:399–404. 10.1017/S0007114515004638 26619904

[78] James PE, Willis GR, Allen JD, Winyard PG, Jones AM. Nitrate pharmacokinetics: taking note of the difference. Nitric Oxide. 2015; 48:44–50. 10.1016/j.niox.2015.04.006 25937621

[79] Wylie LJ, Ortiz de Zevallos J, Isidore T, Nyman L, Vanhatalo A, Bailey SJ, Jones AM. Dose-dependent effects of dietary nitrate on the oxygen cost of moderate-intensity exercise: acute vs. chronic supplementation. Nitric Oxide. 2016; 57:30–39. 10.1016/j.niox.2016.04.004 27093910

[80] Jensen-Urstad K, Johansson J. Gender difference in age-related changes in vascular function. J Intern Med. 2001; 250:29–36. 10.1046/j.1365-2796.2001.00843.x 11454139

[81] Richalet JP, Larmignat P, Poitrine E, Letournel M, Canouï-Poitrine F. Physiological risk factors for severe high-altitude illness: a prospective cohort study. Am J Respir Crit Care Med. 2012; 185:192–98. 10.1164/rccm.201108-1396OC 22071330

[82] Laurent S, Boutouyrie P, Lacolley P. Structural and genetic bases of arterial stiffness. Hypertension. 2005; 45:1050–55. 10.1161/01.HYP.0000164580.39991.3d 15851625

[83] Van Bortel LM, Laurent S, Boutouyrie P, Chowienczyk P, Cruickshank JK, De Backer T, Filipovsky J, Huybrechts S, Mattace-Raso FU, Protogerou AD, Schillaci G, Segers P, Vermeersch S, Weber T, and Artery Society, and European Society of Hypertension Working Group on Vascular Structure and Function, and European Network for Noninvasive Investigation of Large Arteries. Expert consensus document on the measurement of aortic stiffness in daily practice using carotid-femoral pulse wave velocity. J Hypertens. 2012; 30:445–48. 10.1097/HJH.0b013e32834fa8b0 22278144

[84] Pereira T, Maldonado J. Pulse wave analysis reproducibility with the Complior Analyse device: a methodological study. Blood Press Monit. 2018; 23:164–70. 29537992 10.1097/MBP.0000000000000316

[85] Le Roux-Mallouf T, Vibert F, Doutreleau S, Verges S. Effect of acute nitrate and citrulline supplementation on muscle microvascular response to ischemia-reperfusion in healthy humans. Appl Physiol Nutr Metab. 2017; 42:901–08. 10.1139/apnm-2017-0081 28460182

[86] Lévénez M, Garland SJ, Klass M, Duchateau J. Cortical and spinal modulation of antagonist coactivation during a submaximal fatiguing contraction in humans. J Neurophysiol. 2008; 99:554–63. 10.1152/jn.00963.2007 18046002

[87] Lakens D. Calculating and reporting effect sizes to facilitate cumulative science: a practical primer for t-tests and ANOVAs. Front Psychol. 2013; 4:863. 10.3389/fpsyg.2013.00863 24324449 PMC3840331

